# QTL mapping of leukocyte telomere length in American Indians: The Strong Heart Family Study

**DOI:** 10.18632/aging.100600

**Published:** 2013-09-11

**Authors:** Yun Zhu, V. Saroja Voruganti, Jue Lin, Tet Matsuguchi, Elizabeth Blackburn, Lyle G. Best, Elisa T. Lee, Jean W. MacCluer, Shelley A. Cole, Jinying Zhao

**Affiliations:** ^1^ Department of Epidemiology, School of Public Health and Tropical Medicine, Tulane University, New Orleans, LA 70112, USA;; ^2^ Department of Genetics, Texas Biomedical Research Institute, San Antonio, TX 78227, USA;; ^3^ Department of Biochemistry and Biophysics, University of California, San Francisco, CA 94143, USA;; ^4^ Missouri Breaks Industries Research Inc., Timber Lake, SD 7625, USA;; ^5^ Center for American Indian Health Research, University of Oklahoma Health Science Center, Oklahoma City, OK 73104; USA

**Keywords:** leukocyte telomere length, genome-wide linkage scan, quantitative trait loci, American Indians

## Abstract

Telomeres play a central role in cellular senescence and are associated with a variety of age-related disorders such as dementia, Alzheimer's disease and atherosclerosis. Telomere length varies greatly among individuals of the same age, and is heritable. Here we performed a genome-wide linkage scan to identify quantitative trait loci (QTL) influencing leukocyte telomere length (LTL) measured by quantitative PCR in 3,665 American Indians (aged 14 – 93 years) from 94 large, multi-generational families. All participants were recruited by the Strong Heart Family Study (SHFS), a prospective study to identify genetic factors for cardiovascular disease and its risk factors in American Indians residing in Oklahoma, Arizona and Dakota. LTL heritability was estimated to be between 51% and 62%, suggesting a strong genetic predisposition to interindividual variation of LTL in this population. Significant QTLs were localized to chromosome 13 (Logarithm of odds score (LOD) = 3.9) at 13q12.11, to 18q22.2 (LOD = 3.2) and to 3p14.1 (LOD = 3.0) for Oklahoma. This is the first study to identify susceptibility loci influencing leukocyte telomere variation in American Indians, a minority group suffering from a disproportionately high rate of type 2 diabetes and other age-related disorders.

## INTRODUCTION

Telomeres are repetitive DNA sequences and their protective proteins on the distal ends of the chromosomes. They are critical in maintaining genomic stability during mitotic cell proliferation [1, 2]. Telomere length shortens progressively during each round of cell division and declines significantly with age, thus emerging as a valuable biomarker for cellular senescence [2]. Shorter telomere length has been associated with a wide range of age-related disorders, such as dementia [3], cancer [4-6], cardiovascular disease [7, 8], diabetes [8] and Werner syndrome [9] as well as increased mortality. In addition, there is a considerable variation in telomere length among individuals at birth and afterward [10]. However, little is known about the genetic basis underlying the interindividual variability in telomere length.

Leukocyte telomere length (LTL) has been shown to be highly heritable, with heritability estimates ranging from 36% to 84% [10, 11]. Genome-wide linkage analyses have mapped putative loci for LTL onto human chromosomes 3p26.1 [11, 12], 10q26.13 [11], 12p11.2 [13], and 14q23.2 [11], but these loci have not been replicated in independent studies [12, 14]. Genome-wide association studies (GWAS) have also reported genetic variants influencing LTL at or near genes coding for the telomerase RNA component (*TERC)* (3q26.2) [12, 14, 15], oligonucleotide/oligosac-charide-binding fold-containing protein 1 (*OBFC1)* (10q24.33) [14-16], phosphatidylinositol 3-kinase, class 3 (*VPS34/PIK3C3)* (18q12.2), and more recently, conserved telomere maintenance componen1 (*CTC1)* (17p13.1) and zinc finger protein 676 (*ZNF676)* (19p12) [15]. A recent GWAS meta-analysis also identified seven loci, five of which contain candidate genes that are known to be involved in telomere biology [17]. However, these loci have not been convincingly replicated in other populations and inconsistent results were reported among different studies [18]. In addition, common variants in known candidate genes related to telomere maintenance did not exhibit a strong effect on telomere length variation [19]. To date, no study has investigated the genetic determinants of LTL in American Indians. The purpose of this study was to perform a genome-wide linkage scan to localize quantitative trait loci (QTLs) for LTL measured by quantitative PCR in a large population of American Indians participating in the Strong Heart Family Study (SHFS).

## RESULTS

After excluding participants with missing covariate data (N=77) and telomere data (N=1), a total of 3,587 individuals were included in the current analysis. All relative pairs utilized in this study are shown in Table 1. Table 2 shows the characteristics of the study participants according to study center. The mean age of the study participants was 39.9 years old (standard deviation = 17). Women accounted for 60% of the study population. The SHFS participants had a high prevalence of diabetes, especially those from the Arizona center. A high prevalence of cigarette smoking and alcohol consumption was also noted. Age, sex, BMI, study center, and total triglyceride were identified to be significant factors influencing LTL. These covariates accounted for 5.8%, 15.9%, and 22.9% interindividual variability in LTL for participants from OK, AZ and DK, respectively. In analysis using all samples, the proportion of variance due to covariates was 14.1%.

**Table 1:** Relative pairs utilized in this study

Relationship	All	AZ	DK	OK
Identical sib pairs	4	1	2	1
Parent –offspring	2808	856	1017	935
Siblings	2659	880	950	829
Grandparent-grandchild	1055	361	368	326
Avuncular	6662	1983	2549	2130
Half siblings	938	310	317	311
Grand avuncular	2996	783	1223	990
Half avuncular	1359	432	416	511
First cousins	8997	2864	3418	2715
First cousins, once removed	12379	3768	4815	3796
Half first cousins	1303	531	359	413
First cousins, twice removed	1443	353	360	730
Half first cousins, once removed	1594	815	263	516
Second cousins	7003	2201	2972	1830
Second cousins, once removed	2670	867	944	859
Third cousins	929	180	575	174
Half second cousins	792	428	133	231
Half second cousins, once removed	144	62	20	62
Second cousins, twice removed	147	-	144	3
Third cousins, once removed	123	-	120	3
Half third cousins	4	4	-	-
Other relationships	2909	634	1129	1146
Total	58,918	18,313	22,094	18,511

**Table 2:** Basic characteristics of the Strong Heart Family Study participants by center

	Arizona	Oklahoma	Dakotas
Number of subjects	1208	1189	1190
Age (years)	37.02±15.84	43.63±17.35	39.07±17.08
Female sex (%)	62.6%	58.8%	59.0%
Current alcohol drinker (%)	59.3%	47.5%	66.3%
Current smoker (%)	25.5%	33.3%	42.5%
Type 2 diabetes (%)	33.1%	20.6%	14.2%
Body mass index (mean ± SD, kg/m^2^)	35.43±8.79	31.11±6.9	30.19±6.83
Total cholesterol (mean ± SD, mg/dL)	174.35±34.33	185.67±37.26	181.85±39.09
High-density lipoprotein (mean ± SD, mg/dL)	48.55±14.11	52.96±15.4	50.79±13.84
Total triglyceride (mean ± SD, mg/dL)	169.54±134.85	172.4±171.53	161.2±202.1
eGFR (mean±SD, ml/min/1.73m^2^)	111.86±32.37	92.86±23.88	95.57±24.84
Low-density lipoprotein (mean ± SD, mg/dL)	93.79±25.93	99.8±30.4	100.68±31.11
Telomere length (mean ± SD, T/S ratio)	0.95±0.21	0.97±0.22	1.02±0.26

Multivariate-adjusted heritability of LTL was estimated to be 62.4%, 54.8% and 50.9% for participants from OK, AZ and DK, respectively, with an average heritability of 55.6% for all subjects (Table 3).

**Table 3:** Multivariate-adjusted heritability of LTL in SHFS

Center	Heritability (SE)	P value	Proportion of variance due to covariates
All	0.556 (0.03)	1.4 × 10!!^−138^	0.141
Arizona	0.548 (0.06)	3.8 × 10^−32^	0.159
Dakotas	0.509 (0.05)	8.0 × 10^−41^	0.229
Oklahoma	0.624 (0.05)	4.3 × 10^−70^	0.058

Adjusting for age, sex, center, BMI and total triglyceride

**Figure 1:**
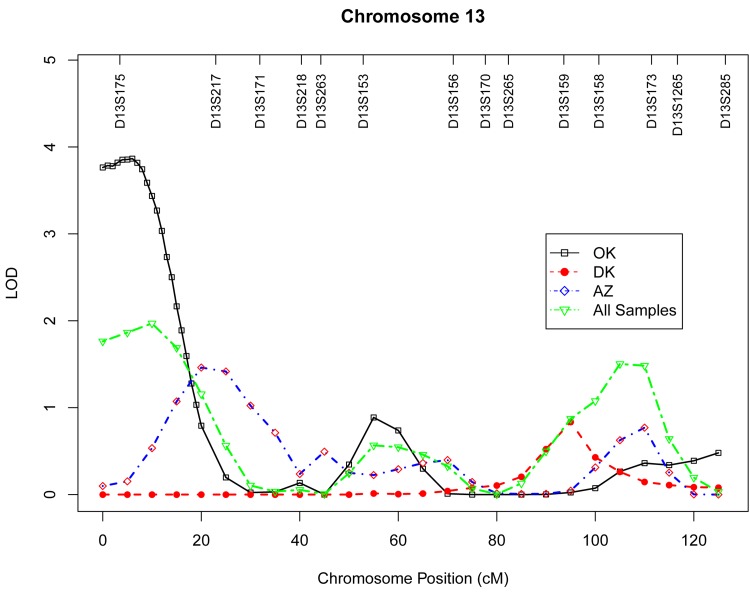
Multipoint LOD scores on chromosome 13 for log-transformed leukocyte telomere length for each center and combined samples. Model was adjusted for age at enrollment, sex, BMI, and total triglyceride. The analysis for combined sample additionally adjusted for study center.

**Figure 2:**
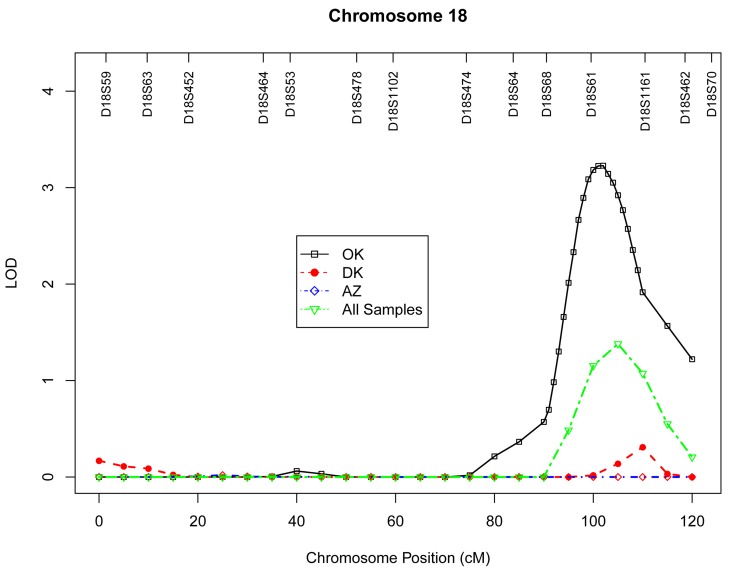
Multipoint LOD scores on chromosome 18 for log-transformed leukocyte telomere length for each center and combined samples. Model was adjusted for age at enrollment, sex, BMI, and total triglyceride. The analysis for combined sample additionally adjusted for study center.

**Figure 3:**
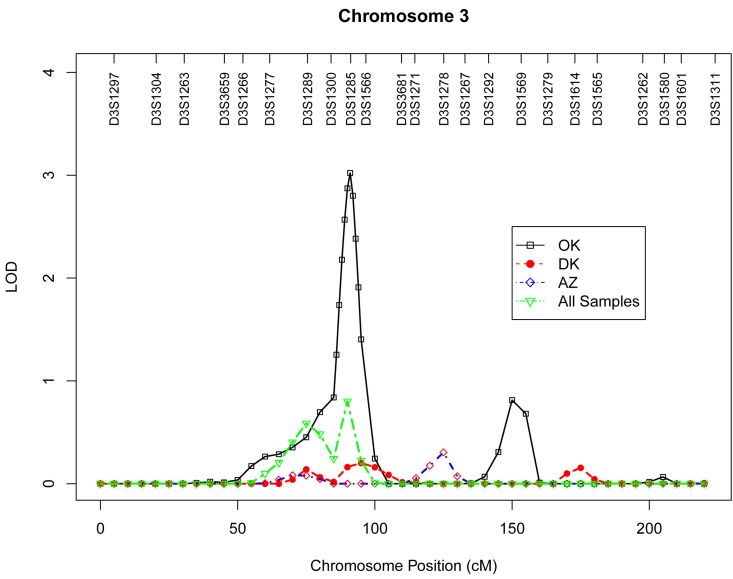
Multipoint LOD scores on chromosome 3 for log-transformed leukocyte telomere length for each center and combined samples. Model was adjusted for age at enrollment, sex, BMI, and total triglyceride. The analysis for combined sample additionally adjusted for study center.

Table 4 presents the results of multipoint genome-wide linkage analyses for LTL, including LOD scores and their locations for all linkage peaks with LOD score ≥ 2.0. Among participants from the Oklahoma population, we identified significant evidence for linkage on chromosome 13 at 6 cM (marker D13S175, LOD score 3.9, Figure 1), chromosome 18 at 101 cM (marker D18S61, LOD score 3.2, Figure 2), chromosome 3 at 91cM (marker D3S1285, LOD score 3.0, Figure 3), and suggestive evidence for linkage on chromosome 20 at 100 cM (marker D20S171, LOD score 2.0) and chromosome 1 at 241 cM (marker D1S2800, LOD score 2.0). A suggestive linkage was also observed on chromosome 7 at 141cM (marker D7S640, LOD score 2.4) in the Arizona pedigrees. No significant or suggestive linkage (LOD score ≥ 2.0) was observed in the Dakota center. Supplementary Figures 1-4 display genome-wide linkage results for all chromosomes according to study center and all centers combined.

## DISCUSSION

In this study, we demonstrated that LTL in American Indians has a strong genetic component, with heritability estimates ranging from 51% to 62%. A genome-wide linkage scan identified significant evidence of linkage for LTL on chromosomes 13q12.11, 18q22.2 and 3p14.1 in the Oklahoma population. In addition, we observed suggestive evidence for linkage on chromosome 1q42.2 and 20q13 from Oklahoma families, and one suggestive linkage on chromosome 7q33 in the Arizona population. No significant or suggestive evidence of linkage was obtained in the Dakota pedigrees. There is no overlap between observed linkage peaks of different centers, suggesting potential genetic heterogeneity among American Indians from different geographic regions.

The strongest evidence of linkage for LTL in our genome-wide scan was localized to chromosome 13q12 in the Oklahoma population. This region has not been previously reported to harbor loci affecting interindividual variation in LTL in any ethnic groups, thus may represent a novel genetic locus influencing LTL. The one-LOD unit support interval (8.8 Mb) of this linkage signal contains over 50 annotated genes. Among these, two genes could represent promising candidate genes for LTL in American Indians. One is the well-known aging gene *Klotho (KL)*, which is located ~ 10 Mb downstream from the peak 13q LOD score. This gene encodes a type-I membrane protein and functions as an ageing-suppressor gene [20]. Overexpression of this gene extended life span in mice, and *klotho*-deficient mouse (*klotho* −/−) manifested a wide range of aging-related phenotypes, such as short life span, atherosclerosis, and osteoporosis [21-23]. In human population studies, genetic variants in the *KL* gene have been associated with longevity [24] and several age-related disorders, such as cardiovascular disease and its associated risk factors [24, 25] and cognitive function [26], all of which are consistent with its association with life span [21]. Another possible candidate gene located in this 13q region is poly (ADP-ribose) polymerase family, member 4 (*PARP4*) [27]. The *PARP* enzymes recognize DNA strand damages, and DNA binding by *PARP* controls telomere length and chromosomal stability by triggering its own release from DNA ends. Telomeres are the terminal DNA structure of chromosomes and are, therefore, potential targets of *PARP*. Mice lacking *PARP* displayed telomere shortening and chromosomal instability, lending further support for an important role of *PARP* in telomere maintenance [28, 29].

Apart from *KL* and *PARP4*, the 13q linkage peak also includes known candidate genes for inflammation, e.g., arachidonate 5-lipoxygenase-activating protein (*ALOX5AP*), and cancer, e.g., breast cancer 2 early onset (*BRCA2*), all of which may be involved in the aging process. In a recent GWAS meta-analysis, the gene encoding zinc finger protein 676 (*ZNF676*) was related to the regulation of human telomere homeostasis [15]. Interestingly, several genes encoding zinc finger proteins, such as zinc finger, DHHC-type containing 20 (*ZDHHC20)*, zinc finger, MYM-type 2 (*ZMYM2)*, and zinc finger, MYM-type 5 (*ZMYM5)*, are also located within the 13q linkage region identified in our study. The possible role of these zinc finger proteins in telomere maintenance warrants further research.

We also identified evidence for linkage on chromosome 18q22.2 in the gene region of docking protein 6 (*DOK6*), a member of the DOK family of intracellular adaptors that play an important role in RET (rearranged during transfection) signaling cascade [30]. Activated RET signaling causes phosphorylation of key docking tyrosines that bind to several adaptor proteins, resulting in the activation of downstream signal transduction pathways [31], thereby controls key cellular processes, such as cell proliferation, differentiation, and survival [32]. Aberrant RET signaling has been associated with papillary thyroid carcinoma, multiple endocrine neoplasia types 2 syndromes, and Hirschsprung's disease [33]. Genetic defects in genes encoding docking proteins have the potential to cause abnormal interaction with the RET signaling, which in turn may result in aging neurons and contribute to aging-related disorders such as Parkinson's disease [34] and Alzheimer's disease [35].

In a previous GWAS for LTL measured by Southern blot analysis, two SNPs (rs2162440 and rs7235755) on chromosome 18q12.2 were significantly associated with telomere length in the gene region of *VPS34/PIK3C3* in Caucasians [36]. Our linkage signal on chromosome 18q22.2 is ~28Mb downstream of this VPS gene region. Given the relatively large map distance between these two regions, it is uncertain whether these two loci belong to a same genetic locus influencing telomere variation in different populations. It is also possible that the *VPS* locus in Caucasians and the *DOK6* locus in American Indians may represent a long-distance *cis*-regulatory element influencing telomere variation.

Another linkage peak identified in our genome-wide scan was located on chromosome 3p14.1 in the gene region of *ADAMTS9* (*ADAM* metallopeptidase with thrombospondin type 1 motif, 9). As a member of the *ADAMTS* family, *ADAAMTS9* has been implicated in proteoglycan cleavage, organ shape control during development, and angiogenesis inhibition. Genetic polymorphisms in the *ASAMTS9* gene have been associated with body fat distribution [37, 38], diabetes[39] and Alzheimer's disease [40]. Previous studies reported association and replication of genetic variants in the telomerase RNA component *(TERC)* gene, located on 3q26, with telomere length variation [12, 14], but the genomic region we identified in American Indians localizes on the short arm of chromosome 3, and thus may represent a novel locus for LTL.

Except for the above-mentioned results, we also observed several loci with marginal evidence of linkage. Although these signals do not meet the genome-wide significance threshold, these genomic regions may still provide valuable information that is worthy of further investigation.

The major strength of this study includes the large, multi-generational pedigrees with well-characterized phenotypes including demographic, clinical and environmental information. The lack of overlap for linkage regions from different study centers further highlights the potential differences in genetic architecture between American Indians from diverse geographic regions. However, the genetic background of American Indians is likely to be more homogeneous than other population-based studies from urban areas.

In summary, we identified strong evidence for novel genetic loci affecting variation in leukocyte telomere length on chromosomes 13q12, 18q22.2 and 3p14.1 in American Indians who suffer from high rates of diabetes and cardiovascular disease. Several other loci with suggestive linkage were also localized. Our findings are independent of adjustments for multiple covariates, including age at enrollment, sex, center, BMI and total triglyceride, suggesting that these factors may not contribute to the observed linkage signals for telomere length. Our linkage results, coupled with plausible biological functions of the potential candidate genes related to aging, such as *Klotho, PARP4, DOK6*, and *ASAMTS9*, make these genomic regions good candidates for further investigation of causal variants influencing LTL in this minority population. Future research to fine map these candidate regions and to determine causal variants, including rare variants and structural variants, will provide valuable information on telomere biology and aging-related disorders.

## METHODS

The Strong Heart Family Study (SHFS) is a multicenter, family-based prospective study designed to identify genetic factors for cardiovascular disease (CVD), diabetes and their risk factors in American Indians. The study was initiated in 1998 and has examined 3,665 individuals (aged 14 to 93 years) from 94 multi-generational families residing in Arizona (AZ), North and South Dakota (DK) and Oklahoma (OK). *Participants* were *followed up about every four to* five years to collect information on morbidity and mortality for CVD, diabetes and associated risk factors. At each visit, study participants underwent a clinical examination including a personal interview and physical examination. Information on demographic factors, socioeconomic status, medical history, medication use, and lifestyle factors was collected by personal interview using standard questionnaires. A physical examination was conducted, and fasting blood samples were collected for laboratory tests, including fasting glucose, glycosylated hemoglobin, insulin, lipids, lipoproteins, and inflammatory biomarkers as well as a 75-gram glucose tolerance test. The study design and methods of the SHFS have been reported previously [41, 42]. The SHFS protocol was approved by the participating tribes and Institutional Review Boards of the Indian Health Service and the participating institutions. All participants gave informed consent. All SHFS participants with complete genotype and telomere data are included in the current investigation.

### Measurement of leukocyte telomere length (LTL)

Genomic DNA from peripheral blood was isolated according to standard protocols. Leukocyte telomere length (LTL), as measured by T/S ratio, was performed by Dr. Elizabeth Blackburn's laboratory at the University of California San Francisco using a high-throughput telomere length assay system. Primers for the telomere polymerase chain reaction (PCR) (T runs) are *tel1b* [5‘-CGGTTT(GTTTGG)_5_GTT-3’] with a final concentration of 100 nM, and *tel2b* [5‘-GGCTTG(CCTTAC)_5_CCT-3’] with a final concentra-tion of 900 nM. The primers for the single-copy gene (human β-globin) PCR (S runs) are *hbg1* [5' GCTTCTGACACAACTGTGTTCACTAGC-3'] at a final concentration of 300 nM, and *hbg2* [5'-CACCAACTTCATCCACGTTCACC-3'] at a final concentration of 700 nM. All primers were purchased from the Integrated DNA Technologies (Coralville, Iowa) *in a standard desalted form*.

The telomere length assay determines the ratio of telomeric product/single copy gene (T/S) obtained using quantitative PCR (qPCR) according to protocols described previously [43, 44]. The rationale of this method is that the longer the telomeres are in each sample, the more PCR product will be generated in PCR reactions using primers specific for the telomeric DNA. This can be quantified by qPCR using a serially diluted standard DNA and the standard curve method. To normalize the quantity of the input DNA, a single copy gene was amplified in parallel as well. The ratio of the telomeric product versus the single copy gene reflects the average length of the telomeres. The qPCR amplification curves were analyzed by the Roche LightCycler software (Roche Applied Science, Penzberg, Germany), which uses the second derivative maximum method to determine the values of crossing points (Cp's). All standard serial dilution Cp's from all 182 qPCR runs were averaged to form the reference set of Cp's to which all runs were normalized. This was done for the S runs and T runs separately. The average PCR efficiencies of S and T runs were 95% and 84%, respectively, and the Cp values were scaled accordingly. Each sample was assayed three times, each time as triplicate wells in the 384 well assay plate. T/S ratio was calculated by dividing the mean of the T concentration and the S concentration for each of the runs. Three of these T/S ratios were averaged, and standard deviation and percent of coefficient variation (%CV) were calculated. In cases where the %CV were larger than 7%, an S or T Cp value that reduced the %CV most if removed was discounted. The T/S ratios were normalized to the mean of all samples and reported.

*For quality control, seven* control DNA samples from various cancer cell lines were included in each assay plate. These control samples allowed us to create standard curves, which were then integrated into a composite standard curve used for T and S concentration calculations. In our study, *4.1% of the total sample was run in duplication and telomere length of the* replicate *samples* were *significantly correlated* (*r* = 0.95, p<0.0001). T*he* average inter-assay and intra-assay CV was 2.5% and 4.4%, respectively. Lab technicians were blinded to the knowledge of clinical data.

### Measurements of risk factors

Body weight (kg) and height (cm) were measured when participants wore light clothes and no shoes by trained research staff. Body mass index (BMI) was calculated by dividing weight in kilograms by the square of height in meters. Waist circumference (WC) was measured at the level of the umbilicus while the participant was supine. Hip circumference was measured at the level of widest circumference over greater trochanters with the legs close together. Waist/hip ratio (WHR) was calculated as waist circumference divided by hip circumference.

Cigarette smoking was assessed *via* questionnaire and participants were grouped as smokers (current plus former smokers) and compared to never smokers. Participants were categorized into current drinkers, former drinkers and never drinkers based on their history of alcohol consumption. Physical activity was assessed by the mean number of steps per day calculated by averaging the total number of steps recorded each day during a 7-day period. Hypertension is defined as blood pressure levels of 140/90 mm Hg or higher or use of antihypertensive medications. According to the 1997 American Diabetes Association (ADA) criteria [45] diabetes was defined as fasting plasma glucose ≥7.0 mmol/L or receiving insulin or oral hyperglycemic treatment. Impaired fasting glucose (IFG) was defined as a fasting glucose of 6.1-7.0 mmol/L. Fasting glucose <6.1 mmol/L is defined as normal.

### Genotyping

The procedures for genotyping in the SHFS have been described previously [46]. In brief, genomic DNA was isolated from fasting blood samples. Genotype data for ~ 400 microsatellite markers (spaced at approximately 10 cM intervals) were generated using the ABI PRISM Linkage Map Set-MD10 Version 2.5 (Applied Biosystems, Foster City, CA). Pedigree relationships were verified using the pedigree relationship statistical tests package,[47] which employs likelihood-based inference statistics for genome-wide identify-by-descent (IBD) allele sharing. Mendelian inconsistencies were detected using Sim Walk2 [48]. Marker allele frequencies were estimated from all individuals using computerized algorithms. With these screening, less than 1% of all genotypes were excluded from analysis. The chromosomal map used in this study was based on marker locations reported in DeCode Genetics [49].

### Quantitative genetic analysis

Prior to analysis, telomere length was log-transformed to improve normality. Backward stepwise linear regression was used to choose the most significant covariates associated with telomere length. The following variables were included in the full model for selection: age at enrollment, sex, study center, BMI, fasting glucose, fasting insulin, systolic blood pressure, smoking status, alcohol consumption, physical activity, lipids and socioeconomic status. Only variables significantly associated with telomere length were included in the final statistical model for linkage analysis.

Heritability was estimated using maximum likelihood variance components decomposition-method by partitioning the total phenotypic variance in LTL into additive genetic and environmental components [50]. An extension of the variance components decomposition method was used to localize quantitative trait loci (QTL) influencing the variation in LTL. Both heritability and genome-wide linkage analyses were conducted using the computer program, sequential oligogenic linkage analysis routines (SOLAR), version 4.0 [50]. This approach takes into account the identity-by-descent (IBD) relationship matrix estimated using the LOKI package [51], which employs a Markov chain Monte Carlo stochastic procedure by computing the IBD allele sharing at points throughout the genome conditional on the genotype information available at neighboring loci. Fine-mapping at 1cM intervals was performed on all points around locations having a LOD score higher than 0.5. All analyses were first conducted separately for each study center, and then combined across all three centers.

## SUPPLEMENTARY FIGURES


