# Supplementary material for: QTL mapping of leukocyte telomere length in American Indians: The Strong Heart Family Study
Source: Aging (Albany NY). 2013 Sep 11;5(9):704–16. doi: 10.18632/aging.100600 (PMC3808702; doi:10.18632/aging.100600)
Supplement: Supplementary file 1 [file aging-05-704-s001.pdf]

48. Sobel E and Lange K. Descent graphs in pedigree analysis: applications to haplotyping, location scores, and marker-sharing statistics. Am J Hum Genet 1996; 58:1323-1337.
49. Kong A, Gudbjartsson DF, Sainz J, Jonsdottir GM, Gudjonsson SA, Richardsson B, Sigurdardottir S, Barnard J, Hallbeck B, Masson G, Shlien A, Palsson ST, Frigge ML, Thorgeirsson TE, Gulcher JR, and Stefansson K. A high-resolution recombination map of the human genome. Nat Genet 2002; 31:241-247.
50. Almasy L and Blangero J. Multipoint quantitative-trait linkage analysis in general pedigrees. Am J Hum Genet 1998; 62:1198-1211.
51. Heath SC. Markov chain Monte Carlo segregation and linkage analysis for oligogenic models. Am J Hum Genet 1997; 61:748-760.

SUPPLEMENTARY FIGURES

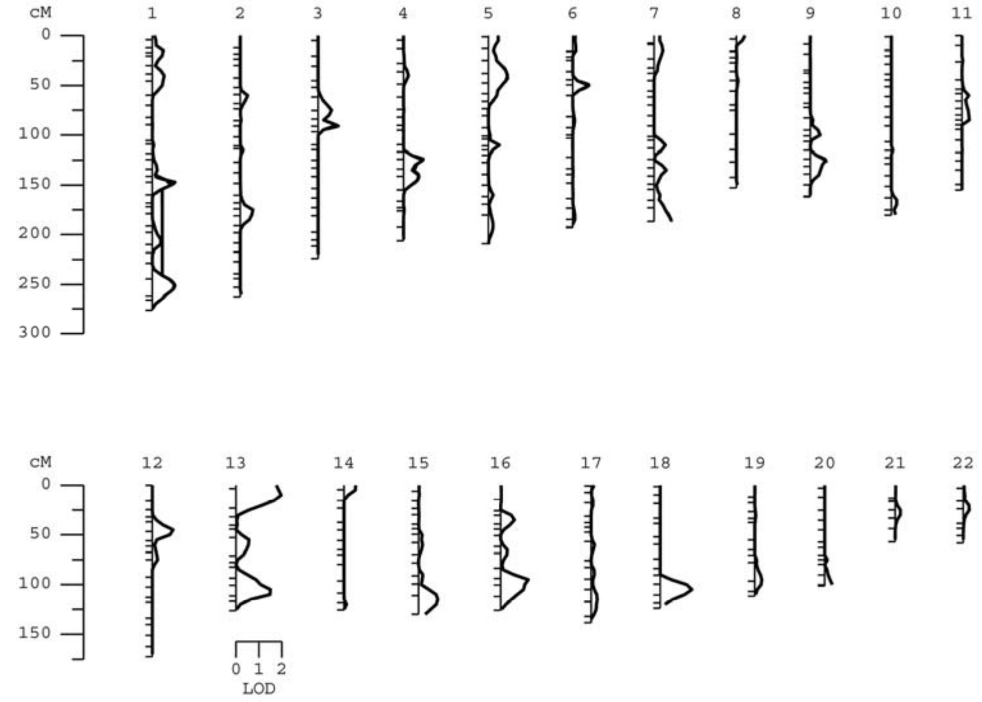

Supplementary Figure 1. Genome-wide multi-point linkage results of log-transformed leukocyte telomere length in combined samples from all three centers. Model was adjusted for age at enrollment, sex, BMI, total triglyceride and study center.

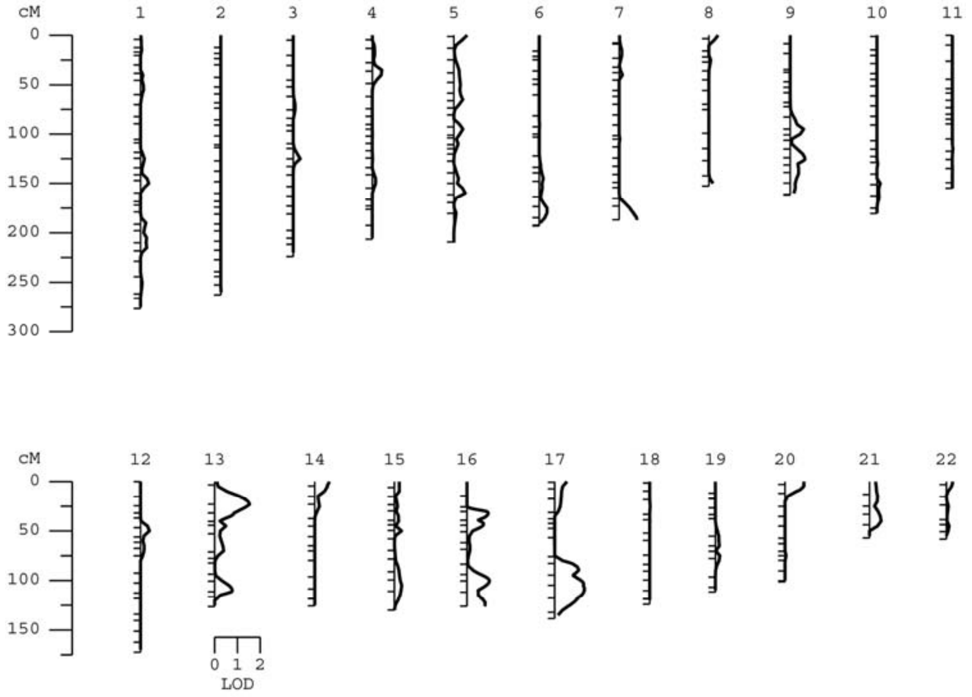

Supplementary Figure 2. Genome-wide multi-point linkage results of log-transformed leukocyte telomere length in Oklahoma center. Model was adjusted for age at enrollment, sex, BMI and total triglyceride.

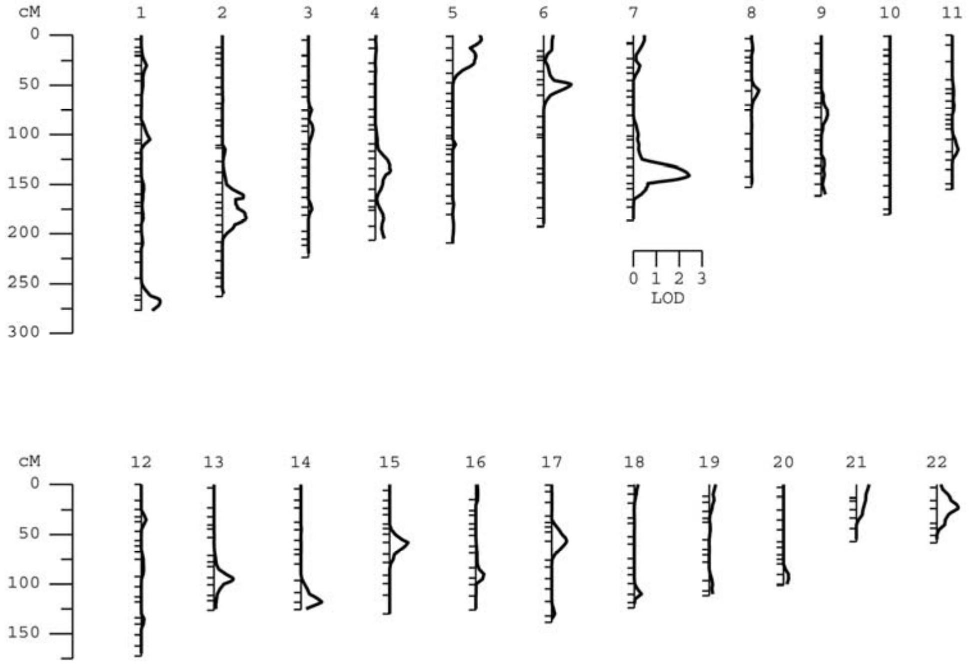

Supplementary Figure 3. Genome-wide multi-point linkage results of log-transformed leukocyte telomere length in Arizona center. Model was adjusted for age at enrollment, sex, BMI and total triglyceride.

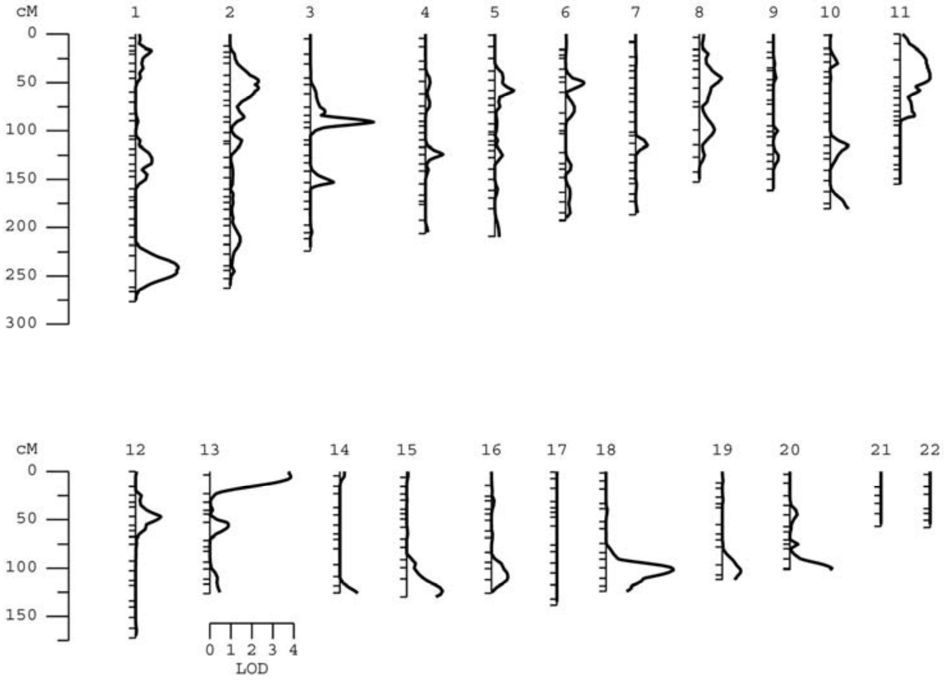

Supplementary Figure 4. Genome-wide multi-point linkage results of log-transformed leukocyte telomere length in Dakota center. Model was adjusted for age at enrollment, sex, BMI and total triglyceride.
